# Antimicrobial Activity of Selected Phytochemicals against *Escherichia coli* and *Staphylococcus aureus* and Their Biofilms

**DOI:** 10.3390/pathogens3020473

**Published:** 2014-06-18

**Authors:** Joana Monte, Ana C. Abreu, Anabela Borges, Lúcia Chaves Simões, Manuel Simões

**Affiliations:** 1 LEPABE, Department of Chemical Engineering, Faculty of Engineering, University of Porto, Rua Roberto Frias, s/n, 4200-465 Porto, Portugal; E-Mails: joana.cmonte@gmail.com (J.M.); anabreu@fe.up.pt (A.C.A.); apborges@utad.pt (A.B.); luciachaves@deb.uminho.pt (L.C.S.); 2 CIQUP, Department of Chemical and Biochemical, Faculty of Sciences, University of Porto, Rua Campo Alegre 687, 4169-007, Porto, Portugal; 3 CECAV, University of Trás-os-Montes e Alto Douro, Quinta de Prados, Apartado 1013, 5001-801 Vila Real, Portugal; 4 CEB, Center of Biological Engineering, University of Minho, Campus de Gualtar, 4710-057 Braga, Portugal

**Keywords:** antimicrobial activity, biofilms, quorum-sensing inhibition, phytochemicals, resistance, synergism

## Abstract

Abstract Bacteria can be resistant to multiple antibiotics and we are fast approaching a time when antibiotics will not work on some bacterial infections. New antimicrobial compounds are urgently necessary. Plants are considered the greatest source to obtain new antimicrobials. This study aimed to assess the antimicrobial activity of four phytochemicals—7-hydroxycoumarin (7-HC), indole-3-carbinol (I3C), salicylic acid (SA) and saponin (SP)—against *Escherichia coli* and *Staphylococcus aureus*, either as planktonic cells or as biofilms. These bacteria are commonly found in hospital-acquired infections. Some aspects on the phytochemicals mode of action, including surface charge, hydrophobicity, motility and quorum-sensing inhibition (QSI) were investigated. In addition, the phytochemicals were combined with three antibiotics in order to assess any synergistic effect. 7-HC and I3C were the most effective phytochemicals against *E. coli* and *S. aureus*. Both phytochemicals affected the motility and quorum-sensing (QS) activity, which means that they can play an important role in the interference of cell-cell interactions and in biofilm formation and control. However, total biofilm removal was not achieved with any of the selected phytochemicals. Dual combinations between tetracycline (TET), erythromycin (ERY) and ciprofloxacin (CIP) and I3C produced synergistic effects against *S. aureus* resistant strains. The overall results demonstrates the potential of phytochemicals to control the growth of *E. coli* and *S. aureus* in both planktonic and biofilm states. In addition, the phytochemicals demonstrated the potential to act synergistically with antibiotics, contributing to the recycling of old antibiotics that were once considered ineffective due to resistance problems.

## 1. Introduction

Resistance to antibiotics is one of the biggest problems that faces public health [1,2]. This problem is a natural consequence of the adaption of infectious pathogens to antimicrobials used in several areas, including medicine, food animals, crop production and disinfectants in farms, hospital and households [3,4,5,6]. Bacteria have developed resistance to all known antibiotics and, as so, the economic burden associated with these multidrug-resistant bacteria is high.

In order to find novel antimicrobial agents with new modes of action, plants have been explored as sources for the identification of new and effective antimicrobials. Plants are an important source of antimicrobial products, most of them with efficiency against diverse organisms including fungi, yeasts and bacteria, insects, nematodes and other plants [7]. Phytochemicals are able to inhibit peptidoglycan synthesis, damage microbial membrane structures, modify bacterial membrane surface hydrophobicity and also modulate quorum-sensing (QS) [8].

While planktonic bacteria are already resistant to many antimicrobials, in biofilms this resistance can increase several times. Biofilm may be formed on a variety of surfaces including living tissues, indwelling medical devices, contact lenses, *etc.* [9]. Biofilms constitute a threat in the clinical environment by acting as pools of multidrug resistant pathogenic bacteria. Consequently, in order to prevent biofilm formation, several studies have been performed to find antimicrobial agents that affect the viability of bacteria in biofilms. Natural products from plants have been shown to influence microbial biofilm formation [8,10,11,12,13].

Diverse mechanisms allow microorganisms to come into closer contact with a surface, attach to it, promote-cell interactions and grow as a 3-D structure [14]. Maximum attachment depends upon high surface free energy or wettability of surfaces. Surfaces with high surface free energies are more hydrophilic and generally show greater bacterial attachment than hydrophobic surfaces [9]. The properties of cells, including cell surface hydrophobicity, presence of fimbriae and flagella, and production of extracellular polymeric substances (EPS) also influence the attachment of microbial cell [15]. QS, related with cell-cell signaling, play a role in cell attachment and detachment from biofilms [16]. QS regulates a wide number of physiological activities, such as motility, conjugation, competence, sporulation, virulence and biofilm formation. The QS signal molecules may alter distribution of bacterial species in the biofilm, alter protein expression, introduce new genetic trait and incorporate bacteria in biofilm [15].

The purpose of this study was to assess the antimicrobial efficacy of selected phytochemicals (7-hydroxycoumarin (7-HC), indole-3-carbinol (I3C), salicylic acid (SA) and saponin (SP)) against *E. coli* and *S. aureus* planktonic cells. These phytochemicals belongs to two different chemical classes: phenolics and glucosinolates. These chemicals are characterized for their antimicrobial, antifungal, antioxidant and anticancer activities [7,17,18,19,20,21]. Moreover, the effects of these phytochemicals were assessed on biofilm control. The phytochemicals were placed in contact with 24 h old biofilms of *E. coli* and *S. aureus* to verify if they were able to promote biomass removal and/or metabolic inactivation. The mode of action of the selected phytochemicals on planktonic cells and in the early stages of biofilm formation was also evaluated by analyzing the effects on the surface charge, free energy of adhesion, bacterial motility and quorum-sensing inhibition (QSI). Additionally, since co-therapies between antimicrobials are being extensively applied in the clinical setting in order to increase the pharmacological activity or decrease the resistance behavior of bacteria, the phytochemicals were combined with three antibiotics (ciprofloxacin (CIP), tetracycline (TET) and erythromycin (ERY)) against several resistant *S. aureus* strains (*S. aureus* RN4220, SA1199B and XU 212) owning different efflux pumps.

## 2. Results and Discussion

### 2.1. Evaluation of Antimicrobial Activity of Phytochemicals

The minimum inhibitory concentration (MIC) and minimum bactericidal concentration (MBC) of 7-HC, I3C, SA and SP were assessed for *E. coli* and *S. aureus* (Table 1). SP was the unique compound that had neither detectable MIC nor MBC for concentrations lower than 3200 µg/mL. The 7-HC and I3C were the most effective compounds against both bacteria, since they presented the lowest values of MIC (800 µg/mL for both phytochemicals against *E. coli*; 200 and 400 µg/mL for 7-HC and I3C, respectively, against *S. aureus*). Considering that most reports about natural products and extracts describe MIC values over 1000 µg/mL, which has little relevance for clinical application [22], the results obtained with 7-HC and I3C are considered relevant. Therefore, in this sense and considering that phytochemicals are routinely classified as antimicrobials on the basis of susceptibility tests that produce inhibitory concentration in the range of 100–1000 µg/mL, these compounds could be classified as antimicrobial [23,24]. Concerning the MBC, I3C seems to be the most effective phytochemical against both bacteria. The MBC for 7-HC was not detected for both bacteria, until the maximum value tested (10,000 µg/mL). The MIC/MBC values obtained are in the range of those described in other studies. In comparison with previous studies with phytochemical products, essential oils of *Laportea aestuans* in that a methyl ester of SA, methyl salicylate, as the main compound, showed inhibitory potential especially at 200 mg/mL, against various microorganisms, including *E. coli* and *S. aureus* [25]. In a study performed by Hassan and collaborators [26], saponin-rich extracts from guar meal and quillaja exhibited antibacterial activity against *S. aureus*. Another study showed antibacterial activity of saponin from leaves and bark of *Acacia Arabica* against diarreagenic *E. coli* [27]. Antimicrobial activity of coumarin and several derivatives against *E. coli* and *S. aureus*, was demonstrated by Souza *et al.* [28]. The MIC and MBC values ranging between 500 to 2000 µg/mL, and 1000 to 2000 µg/mL, respectively. Another study reported that 7-HC derivatives had antimicrobial activity against a panel of Gram-negative and -positive bacteria, including *E. coli* and *S. aureus* [29]. Moreover, these authors found that the long-chain of fatty acids esters are related with the efficacy of the 7-HC. An amino-coumarin, 7-amino-4-methylcoumarin, from *Ginkgo biloba*, had broad-spectrum antibacterial activities against *S. aureus*, *E. coli*, *Salmonella enterica* serovar Typhimurium, *Salmonella enteritidis*, *Aeromonas hydrophila*, *Yersinia* sp., *Shigella* sp. and *Vibrio parahaemolyticus* [30]. Strong activity was also obtained by Kuete *et al.* [31], with 7-HC isolated from *Treculia obovoidea* (Moraceae) against some Gram-positive and -negative bacteria.

**Table 1:** MIC and MBC of the selected phytochemicals against *E. coli* and *S. aureus.*

Strains	Phytochemicals (µg/mL)						
	7-HC	I3C	SA	SP
MIC							
*E. coli* CECT 434	800	800	3200	ND
*S. aureus* CECT 976	200	400	1600	ND
MBC							
*E. coli* CECT 434	ND	1600	5000	ND
*S. aureus* CECT 976	ND	800	3200	ND

ND—Not detected.

In a study performed by Borges *et al.* [32], gallic (hydroxybenzoic acid) and ferulic acids (hydroxycinnamic acid) demonstrated antimicrobial activity against *E. coli* and *S. aureus*. In addition, these authors showed that hydroxycinnamic acid was more effective than hydroxybenzoic acid, considering the MIC and MBC values. These results are in accordance with those obtaining in the present study for 7-HC (hydroxycinnamic acid) and SA (hydroxybenzoic acid), relatively to the MIC values. Hydroxycinnamic acids are, generally, antibacterial and less polar than the corresponding hydroxybenzoic acids, due to their propenoid side chain, and this property might facilitate the transport of these molecules through the cell membrane [33,34]. Nonetheless, the values of MBC of SA for the two strains tested were lower than those of 7-HC.

Other authors tested the antimicrobial activity of some glucosinolates and their enzymatic hydrolysis products including I3C, against several Gram-negative (*Acinetobacter baumanii*, *Citrobacter freundii*, *Enterobacter asburiae*, *Enterobacter cloacae*, *Enterobacter hormaechei*, *Escherichia coli*, *Hafnia alvei*, *Klebsiella oxytoca*, *Klebsiella pneumoniae*, *Morganella morganii*, *Proteus mirabilis*, *Pseudomonas aeruginosa*, *S. typhimurium* and *Stenotrophomonas maltophilla*) and –positive bacteria (*Enterococcus faecalis*, *Staphylococcus aureus*, *Staphylococcus saprophyticus*) isolated from the human intestinal tract [35]. The authors found that intact glucosinolates had no effect on any of the Gram-positive or -negative bacteria tested, isothiocyanates were the most effective glucosinolate hydrolysis products (GHP) against both Gram-negative and –positive bacteria, and I3C had only some inhibitory activity against the Gram-positive bacteria.

In our study, the values of MIC and MBC for *E. coli* were higher than those for *S. aureus*. This result was expected because *E. coli* is a Gram-negative bacterium and the presence of an outer membrane (OM) can make it less susceptible to antimicrobials than Gram-positive bacteria [36,37]. Indeed, the Gram-negative bacteria have lipopolysaccharide (LPS) in their OM, which functions as a barrier that slows the penetration of antimicrobials. In these bacteria, the passage through the OM is regulated by the presence of hydrophilic channels (porins) that usually exclude the entry of hydrophobic compounds. Moreover, the OM of these bacteria lacks phosphoglycerides and, hence, the effective channels for hydrophobic diffusion [38,39].

### 2.2. Aspects Underlying the Antibacterial Action and Biofilm Control of Phytochemicals

#### 2.2.1. Surface Charge and Hydrophobicity

It is important to remember that microorganisms have different mechanisms of adhesion and retention, influenced by the substrata, nutrients, ionic strength, pH values and temperatures, and also their phenotype and genotype [40]. The ability of microorganisms to attach to the surfaces is crucial for the beginning of colonization. The process of adhesion of microorganisms to surfaces is very complex, and is affected by many variables. The hydrophobicity and the charge of the cell surface, the presence of bacterial adhesins (e.g., fimbriae, flagella and pili), and particularly the quantity and composition of generated EPS, are the main factors that influence both the rate and degree of microbial adhesion [41]. Indeed, the surface properties of microbial cells have a major impact on adhesion to a substratum. The surface charge of the cells is often determined as its zeta potential, which is measured from the mobility of cells in the presence of an electrical field under well-known conditions (pH and salt concentrations) [42,43]. At physiologic conditions, most of the microorganisms are negatively charged due to the presence of anionic groups, such as carboxyl and phosphate, in their membranes [42]. Table 2 shows the several variations of zeta potential of cells in the presence of the different phytochemicals.

**Table 2:** Zeta potential (mV) results of suspensions of *E. coli* and *S. aureus* exposed to the phytochemicals at their MIC.

	Zeta Potencial (mV)	
	*E. coli* CECT 434	*S. aureus* CECT 976
Control	−13.0 ± 1.4	−29.8 ± 1.3
7-HC	−13.5 ± 1.8	−26.0 ± 6.2
I3C	−21.0 ± 5.7	−27.3 ± 6.3
SA	−1.80 ± 0.3	1.80 ± 0.5
SP	−12.7 ± 1.6	−19.4 ± 2.2

Both bacterial strains present a negative surface charge: −13.0 mV for *E. coli* and −29.8 mV for *S. aureus*. 7-HC and SP have no significant effect in the charge of *E. coli* (*p* > 0.05). In contrast, I3C and SA seem to change the *E. coli* surface charge (*p* < 0.05) for more and less negative values, respectively. The charge of *S. aureus* surface become less negative when exposed to all compounds, but significant alteration was observed when in contact with SA and SP (*p* < 0.05). The first one was able to make the membrane surface positive, while the exposure to SP changed the surface charge of cells to less negative values. Other authors have also studied the effects of phenolic compounds in cell surface charge with the same bacteria [32]. In accordance with our results, they showed that *E. coli* and *S. aureus* has a negative surface charge. The interaction between bacteria and phenolics (gallic and ferulic acids) change the surface charge of cells to less negative values especially for *E. coli*. In the case of *S. aureus*, the zeta potential values were similar to the control. In a study performed by Abreu *et al.* [7], the zeta potential was studied for *E. coli* and *S. aureus* when in contact with a glucosinolate (phenyl isothiocyanate). It was verified that the values of surface charge of the cells become less negative for both bacteria. In our study, this was particularly verified in the case of *S. aureus*.

As mentioned above, the hydrophobicity has been characterized as one important aspect in bacterial adhesion [44]. The hydrophobicity can be calculated through the energy of hydrophobic attraction (∆**G**^TOT^). If ∆**G**^TOT^ < 0 mJ/cm^2^, the interaction between the surface molecules is attractive, which means that molecules have less affinity for water than among themselves, and the surface is considered hydrophobic. If ∆**G**^TOT^ > 0, the surface is considered hydrophilic, and the interaction between the surface molecules is repulsive. Therefore, the more negative the value of ∆**G**^TOT^, more hydrophobic is the surface; and the more positive the value of Δ**G**^TOT^, more hydrophilic is the surface [44,45].

Table 3 presents the results of hydrophobicity obtained for *E. coli* and *S. aureus* cells in the presence of the four phytochemicals. Regarding the several parameters, the apolar component (γ*^LW^*) was particularly changed when *E. coli* is treated with I3C and SP (*p* < 0.05), making their surface less apolar. The same phytochemicals were also able to change the γ*^AB^* component making the cells more polar (*p* < 0.05). However, in the presence of 7-HC and mainly SA, *E. coli* become less polar. The electron donor component increased with the application of I3C and SP and decreased with 7-HC and SP. In the case of *S. aureus*, the treatment with the several phytochemicals did not permit variations on the polarity of surface molecules (*p* > 0.05). Except in the case of SA, the value of γ*^AB^* was lower than the observed in the control, which means that the surface of molecules was less hydrophilic. Regarding their electron donor capacity, 7-HC and SA varied significantly (*p* < 0.05), compared with the values obtained when *S. aureus* was not treated with phytochemicals. These phytochemicals decreased the capacity to donate electrons, *i.e.*, when *S. aureus* was in contact with 7-HC and SA the cell lost its ability to give electrons (γ^−^ decreased).

The ΔG^TOT^ values obtained for *E. coli* and *S. aureus*, before exposure to the phytochemicals, show that they have hydrophilic character (ΔG^TOT^ > 0 mJ/m^2^). It is possible to observe changes in the bacterial membrane physicochemical character of *E. coli* with the application of all compounds particularly with SA and SP (*p* < 0.05). The application of 7-HC, I3C and SP promoted the decrease of hydrophilic properties of *E. coli*. However, with SA the cell surface of this bacterium become more hydrophilic. The opposite effect was observed for *S. aureus* with SA. This phytochemical induced a cell surface hydrophobic character (*p* < 0.05). Similar result was obtained with 7-HC. However, I3C increased bacterial hydrophilic character (*p* < 0.05). In general, the results obtained demonstrated that the selected phytochemicals significantly interact with bacterial surface constituents, modifying their physicochemical properties. In other work [7], the interaction of a GHP, phenyl isothiocyanate, with bacterial cells was assessed. The results demonstrated that the interaction caused an alteration of cell surface hydrophobicity. The interaction with the phytochemical promoted the increase of their hydrophilic character. Even if the cell membrane is the first contact point between the microorganism and the phytochemical, to our knowledge there are no relevant studies on the effects of phytochemicals on microbial surface properties.

**Table 3:** Hydrophobicity (∆**G**^TOT^), and apolar (**γ**^LW^) and polar (**γ**^AB^) components of the surface tension of untreated (control) and phytochemical treated cells. The means ± SDs are illustrated.

Bacteria	Phytochemical	Surface Tension Parameters (mJ/m^2^)				Hydrophobicity (mJ/m^2^)
		γ^LW^	γ^AB^	γ *^+^*	γ *^−^*	∆G^TOT^
*E. coli* CECT 434	Control	33.6 ± 5.0	22.4 ± 5.4	2.6 ± 0.5	52.0 ± 4.8	28.9 ± 7.1
	7-HC	30.7 ± 4.8	20.8 ± 4.7	1.50 ± 0.3	44.9 ± 7.1	21.0 ± 5.1
	I3C	20.2 ± 4.8	37.1 ± 7.3	7.80 ± 1.9	55.8 ± 6.7	20.6 ± 3.8
	SA	31.0 ± 5.2	2.07 ± 6.0	1.50 ± 0.3	59.7 ± 11	37.6 ± 18
	SP	21.0 ± 1.8	40.1 ± 3.1	7.80 ± 1.3	52.1 ± 2.8	19.7 ± 3.3
*S. aureus* CECT 976	Control	35.4 ± 5.4	19.7 ± 4.6	2.00 ± 0.4	53.5 ± 3.8	30.2 ± 3.2
	7-HC	36.2 ± 3.4	21.1 ± 3.8	2.70 ± 0.3	47.8 ± 4.2	22.4 ± 4.8
	I3C	34.5 ± 4.2	20.4 ± 4.8	2.20 ± 0.5	55.4 ± 5.1	32.2 ± 7.3
	SA	37.4 ± 3.0	15.2 ± 3.5	1.50 ± 0.3	44.8 ± 7.7	22.6 ± 5.6
	SP	36.1 ± 4.4	18.3 ± 4.4	2.1 ± 0.5	54.4 ± 1.5	30.4 ± 2.9

ΔG^TOT^ > 0 mJ/m^2^—Hydrophilic; ΔG^TOT^ < 0 mJ/m^2^—Hydrophobic.

Bacterial adhesion to a surface is a complex process that can be influenced by several factors: physicochemical properties of the cell (hydrophobicity and surface charge), material surface properties and environmental factors (temperature, pH, exposure time, concentration of bacteria, chemical treatment or the presence of antimicrobials and fluid flow conditions) [40]. Behind these conditions, biological properties of bacteria also influence the attachment to surface, such as: presence of fimbriae and flagella, and the production of EPS [40].

The polystyrene (PS) microtiter plates are usually used as the standard bioreactor system for adhesion and bacterial biofilm formation. According to the PS physicochemical surface properties, this material was characterized for being hydrophobic and present negative surface charge (∆**G**^TOT^ = −44 mJ/m^2^; zeta potential = −32 ± 2 mV) [40]. Therefore, in order to predict the ability of microorganisms to adhere to PS surfaces, the free energy of interaction between the bacterial surface and the PS surface was assessed according to a thermodynamic approach (Table 4). The thermodynamic theory of adhesion permits the quantification of the free energy of adhesion and predicts the possibility of establishment of an interface between a surface and the microorganism. Analyzing the free energy of adhesion, it is possible to conclude that both *S. aureus* and *E. coli* have no theoretical thermodynamic capacity to adhere to PS (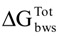 > 0 mJ/m^2^). This was significantly reversed when *S. aureus* was treated with SA (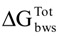 < 0 mJ/m^2^). Besides, a decrease in the free energy of adhesion was found for *S. aureus* and *E. coli* when exposed to 7-HC, and for *S. aureus* exposed to SP. An increase in the 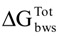 value (less favorable adhesion) was found for *E. coli* when exposed to I3C, SA and SP. A similar effect was observed for *S. aureus* with I3C, where there was an increase in the free energy of adhesion. These distinct values of 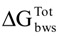 found after exposure to phytochemicals is apparently related to the distinct physicochemical cell surface properties (Table 3). The interaction of the different compounds tested with bacterial cells, appears to depend on the bacteria tested and on the molecule used. Several authors explained that increasing the hydrophobicity can cause an increasing extension of adhesion [46,47,48]. Although it is known that hydrophobicity plays an important role in the adhesion phenomena, the results obtained in this work suggest that this phenomenon is not only influenced by physicochemical surface properties and is governed by other factors. Indeed, the thermodynamic theory of adhesion not do consider the electrostatic interactions and biological aspects of adhesion [44].

**Table 4:** Free energy of adhesion (∆**G**^TOT^_bws_) of *E. coli* and *S. aureus* to polystyrene (PS), with and without exposure to the phytochemicals.

Strain	Phytochemical	Free Energy of Adhesion— 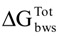 (mJ/m^2^)
*E. coli* CECT 434	Control	4.4 ± 1.2
	7-HC	3.5 ± 0.5
	I3C	15.3 ± 3.3
	SA	10.3 ± 2.1
	SP	13.5 ± 2.1
*S. aureus* CECT 976	Control	5.7 ± 1.2
	7-HC	1.4 ± 0.2
	I3C	6.4 ± 1.2
	SA	−3.2 ± 0.4
	SP	5.4 ± 0.5

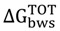
 < 0 mJ/m^2^—thermodynamic favorable adhesion; 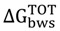 > 0 mJ/m^2^—thermodynamic unfavorable adhesion.

Opposite effects were obtained, when comparing the 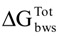 for the Gram-negative (the free energy of adhesion increased), except with 7-HC, and the Gram-positive bacterium (the free energy of adhesion decreased), except with I3C. Therefore, it is expected that I3C, SA and SP can hinder the adhesion of *E. coli* to PS. In addition, I3C may also impair the adhesion of *S. aureus*. In previous studies [49,50,51,52] the anti-adhesive properties of some polyphenolics was described. In these studies, the anti-adhesive tests were performed mainly with *Streptococcus mutans* using glass [52] and saliva-coated hydroxyapatite [49,50,51,52] as adhesion surfaces. Moreover, Borges *et al.* [18,23], demonstrated that some phenolic acids (gallic and ferulic acids) and GHP (allylisothiocyanate and 2-phenylethilisotiocyanate) reduced the potential of adhesion to PS of some pathogenic bacteria, including *E. coli* and *S. aureus*. In other work performed by Luis and coworkers [53], gallic acid was also able to influence the adhesion properties of methicillin-resistant *S. aureus* (MRSA) to PS. Similar results were obtained by Lemos *et al.* [54], with SA against *Bacillus cereus*.

#### 2.2.2. Cell Motility

Motility is one of the most important features in microbial physiology. Bacteria show different ways of motility. Swimming and swarming motilities are two forms of surface flagella-dependent motility existing in *E. coli* [23,54,55]. These types of motility contribute to the virulence of pathogens through adhesion and biofilm formation on biotic and abiotic surfaces [23]. *S. aureus* is a non-flagellated bacterium with a kind of motility defined as sliding or colony spreading [23]. This sliding motility is produced by the expansive forces of a growing colony in combination with reduced surface tension [23].

The phytochemicals at their MIC were tested for their ability to interfere with swimming and swarming motilities of *E. coli*, and sliding motility of *S. aureus*. The results obtained are presented in Table 5. *E. coli* showed an increasing in swimming and swarming motilities over time. Swimming and sliding motility were mostly affected when I3C was added (*p* < 0.05). However, I3C did not influence swarming motility (24 to 72 h). SA was also able to promote a decrease in swimming and sliding motilities but not swarming motility, despite being very low in the first 24 h, increased in the last 48 h. Probably, *E. coli* was able to adapt to SA after a long period of exposure. The application of SP caused an increase in swarming and sliding motilities in the first hours, but after a long period of exposure, both motilities decreased. Finally, 7-HC influenced swarming and swimming motilities but it was not able to change sliding motility. These results may be important because changes in motility can be associated with a reduced ability of bacteria to form biofilms. Likewise, other reports shown that, many natural compounds (extracts or pure products) have capability to interfere with bacterial motility of several microorganisms. Methanolic extracts of *Cuminum cyminum*, which contain methyl eugenol, inhibited swimming and swarming motility of *P. aeruginosa*, *P. mirabilis* and *Serratia marcescens* [56]. Cinnamaldehyde and eugenol from *Cinnamomum cassia* affected the biofilm formation of *E. coli*, through interference with their swimming motility [57].

**Table 5:** Motility results for bacteria with and without phytochemicals. The drop baseline was 6 mm, which was subtracted from the results presented.

Time/Phytochemical	*E. coli* CECT 434		*S. aureus* CECT 976
	Swimming (mm)	Swarming (mm)	Sliding (mm)
**24 h**			
Control	79.0 ± 1.2	8.70 ± 0.6	7.00 ± 0.0
7-HC	7.00 ± 1.0	7.70 ± 1.5	5.00 ± 0.0
I3C	4.70 ± 0.6	7.70 ± 2.9	0.0 ± 0.0
SA	3.30 ± 0.9	2.00 ± 0.9	7.70 ± 0.6
SP	80.0 ± 0.0	56.0 ± 2.0	84.0 ± 0.0
**48 h**			
Control	84.7 ± 0.6	13.7 ± 3.8	8.00 ± 1.0
7-HC	43.3 ± 2.9	8.70 ± 1.2	8.30 ± 0.6
I3C	0.0 ± 0.0	10.0 ± 7.8	0.0 ± 0.0
SA	0.0 ± 0.0	55.0 ± 8.7	0.0 ± 0.0
SP	84.0 ± 0.0	61.7 ± 9.1	56.7 ± 5.8
**72 h**			
Control	84.0 ± 0.0	64.3 ± 7.6	7.70 ± 0.6
7-HC	51.3 ± 2.3	8.30 ± 0.6	8.30 ± 0.6
I3C	0.0 ± 0.0	8.70 ± 5.5	0.0 ± 0.0
SA	0.0 ± 0.0	54.3 ± 6.4	2.00 ± 0.6
SP	84.0 ± 0.0	13.3 ± 3.2	55.0 ± 8.7

In a recent study performed by Borges *et al.* [23], two phenolic acids (gallic acid and ferulic acid), demonstrated potential to inhibit bacterial motility of four pathogenic bacteria (*E. coli*, *P. aeruginosa*, *S. aureus* and *L. monocytogenes*). These authors found similar results with two isothiocyanates (allylisothiocyanate and 2-phenylethylisotiocyanate). In another study, it was found that ferulic acid and SA can inhibit the swimming motility of *Bacillus cereus* and *Pseudomonas fluorescens* [23,54].

A relationship between cells surface motility and biofilm formation has been reported, especially in the case of swarming motility. Both processes, biofilm formation and swarming, require production of flagella and surface polysaccharides [23]. The major role of swarming motility in biofilm development is to promote initial attachment, probably because the force-generating motion helps to overcome electrostatic repulsive forces between bacterium and the substratum, improving the initial interactions between the two surfaces [58]. Therefore, this demonstrates their important function on the early stages of biofilm formation [59,60]. Several authors have reported mutants with altered swarming motility that made it difficult to form biofilm, concluding that they can play a crucial role in biofilm development [58,61].

#### 2.2.3. Quorum-Sensing

QS is a mechanism by which a bacterial population senses its cell density [62]. This mechanism influences bacterial biofilm growth and development and it is related to cell-cell interactions [63]. This cell-cell communication system is dependent on several factors: synthesis, exchange and perception of small signal molecules between bacteria [62]. A disc diffusion assay was performed for QSI screening using the biosensor strain *Chromobacterium violaceum* (CV12472). *C. violaceum* synthesizes the violet pigment as a result of their autoinducers *N*-acyl homoserine lactones (AHLs) based QS systems CviI/CviR (homologs of LuxI/LuxR systems), which sense and responds to autoinducers C6-AHL and C4-AHL [64]. The phytochemicals were tested as QS inhibitors, at several concentrations. Table 6 shows the results obtained.

**Table 6:** Quorum-sensing results for several phytochemicals at different concentrations.

	7-HC			I3C			SA			SP		
Conc. (µg/mL)	QSI pigm.	Inhibition halo (mm)	QS halo (mm)	QSI pigm.	Inhibition halo (mm)	QS halo (mm)	QSI pigm.	Inhibition halo (mm)	QS halo (mm)	QSI pigm.	Inhibition halo (mm)	QS halo (mm)
250	+/−	12	n.d.	+	11	n.d.	+	14	n.d.	+	10	n.d.
500	+/−	11	n.d.	+/−	11	5	+	14	n.d.	+	11	n.d.
750	+/−	10	n.d.	+/−	11	6	+	19	n.d.	+	12	n.d.
1000	+/−	11	7	+/−	11	14	+	16	n.d.	+	14	n.d.
1500	+/−	11	7	+/−	12	15	+/−	14	6	+	9	n.d.
2000	+/−	10	5	−	16	9	−	16	8	+	10	n.d.
3000	+/−	10	8	−	20	5	−	16	8	+	10	n.d.
4000	+/−	12	10	−	20	11	−	15	12	+	10	n.d.
5000	+/−	11	5	−	25	9	−	18	9	+	11	n.d.

(+)—There was formation of purple pigment in the plate; (−)—Purple pigment was not formed in the plate; (+/−)—The pigment formed was clearer. n.d.—halo not detected.

The MIC of the four phytochemicals tested against *C. violaceum* CV12472 ranged from 25 to 3200 µg/mL. This means that phytochemicals were able to inhibit bacterial growth. QS results showed the effects of the phytochemicals in bacterial growth (inhibition halo) and also the effect of phytochemical in QS, through the detection of pigment inhibition (QS halo). Inhibition of pigment production was detected with some phytochemicals at different concentrations. Of all phytochemicals tested, inhibition of pigment production was detected with 7-HC, I3C and SA with zones of pigment inhibition ranging between 5 to 19 mm. No effect on pigment production was observed with SP at the concentrations tested. The I3C was the most effective to inhibit pigment production. At 500 µg/mL of IC3, inhibition was low, but increasing the concentration, the zone of pigment inhibition also increased. Regarding 7-HC and SA at low concentrations, there was no inhibition of pigment production; but for 1000 and 1500 µg/mL of 7-HC and SA, respectively, QSI halos were detected. Although, SP showed antimicrobial activity, it was not possible to observe an effect on pigment inhibition at the concentrations tested. Regarding QS halos, the concentration of I3C, 7-HC and SA influenced significantly the quantity of pigment production. Therefore, inhibition of QS activity by phytochemicals is concentration dependent, as reported by other authors [62,64,65]. The same authors identified one compound (clove oil) able to inhibit pigment production with 19 mm of pigment inhibition zone against *C. violaceum* (CV12472). In addition, cinnamon, peppermint and lavender caused pigment inhibition against the same bacterium [62]. It has been reported that *Tecoma capensis*, *Sonchus oleraceus*, *Pityriasis alba*, *Pinus nigra*, *Jasminum sambac*, *Rosmarinus officinalis*, *Lavandula angustifolia* and *Laurus nobilis* are great sources of antimicrobial compounds and QS inhibitors [66]. Other study demonstrated that isothiocyanates, like allylisothiocyanate, benzylisothiocyanate and 2-phenylethylisothiocyanate, have capacity for QSI by modulation of the activity and synthesis of autoinducers, particularly *N*-acyl homoserine lactones (AHLs), interfering with the QS systems of *C. violaceum* (CV12472) [32]. SA has been described as QS inhibitor for diverse bacteria, including *P. aeruginosa* [54,67].

As QS is an important event that is related with the different steps of bacterial biofilm formation and differentiation, QS inhibitors can be useful in biofilm eradication [16,64]. Moreover, QS inhibitors can help to overcome the selective pressure created by antibiotic use [64]. Therefore, the results obtained in this simple screening assay suggest that the selected phytochemicals can inhibit QS phenomena. In addition, these results reinforce the potential of phytochemical products as QS inhibitors.

The phytochemicals and other compounds that affect QS can act at different levels: inhibition of signal biosynthesis or inhibition of activity of AHL-producing enzymes, enzymatic signal degradation and inhibition of reception signal molecules [62]. More tests would be necessary in order to be conclusive about the specific mechanisms causing this QSI.

### 2.3. Biofilm Control

The effects of the phytochemicals were also tested on *E. coli* and *S. aureus* biofilms. Biofilm eradication is difficult to achieve due to their inherent resistance to antibiotics, biocides and host defenses. There are several mechanisms explaining the resistance of biofilms to antimicrobials [68], which makes difficult to predict the behavior of the biofilm cells. In this study, biofilm formation was performed in sterile 96-well polystyrene microtiter plates for 24 h. After that, biofilms were incubated with phytochemicals at MIC and 5 × MIC for 1 h. The ability of phytochemicals to control (remove and inactivate) 24 h old biofilms was analyzed, based on their effects on biomass and metabolic activity.

**Figure 1:**
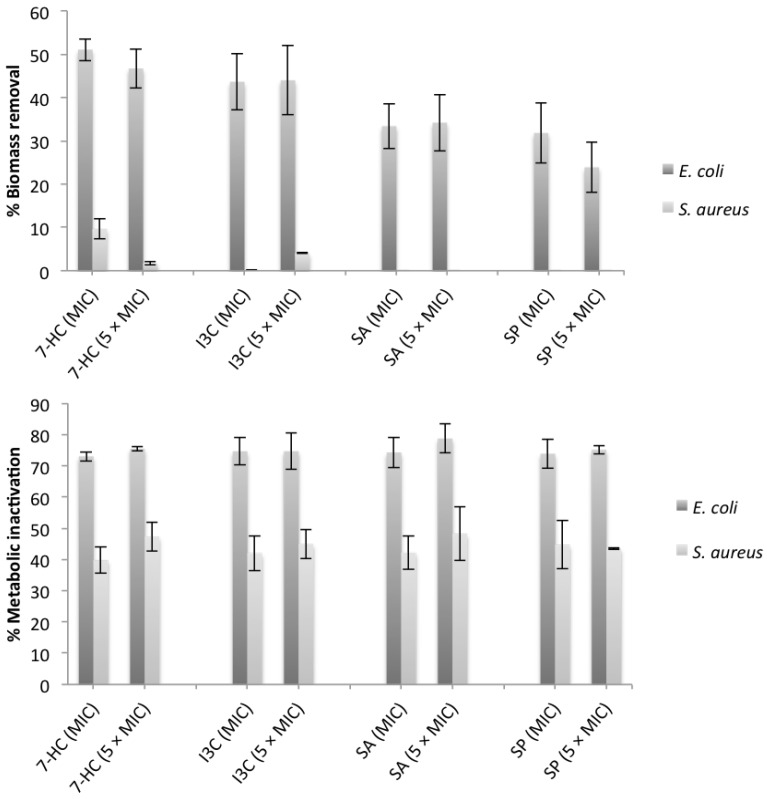
Percentages of removal and inactivation by the selected phytochemicals against *E. coli* and *S. aureus* biofilms.

Figure 1 presents the percentages of biofilm removal and inactivation with the selected phytochemicals at both concentrations. Comparing the values of obtained, it is perceptible that the phytochemical concentration (MIC and 5 × MIC) did not influence the removal and inactivation of the biofilms (*p* > 0.05), for both bacteria. 

The same phytochemical, at the same concentration, caused different behavior in the Gram-negative and -positive bacterium (*p* < 0.05). The percentages of biomass removal and inactivation were always higher for *E. coli* than for *S. aureus* for all the phytochemicals and concentrations tested. Total biofilm removal was not achieved with any of the selected phytochemicals. The highest reduction in biomass was found for *E. coli* with 7-HC.

Despite the greater effect of phytochemicals on biofilms of *E. coli* than on those of *S. aureus*, this result is contradictory to the results obtained with planktonic tests where *E. coli* was less susceptible. In fact, the number of resistance mechanisms in biofilms increase significantly from the planktonic state. Therefore, one cannot infer on the biofilm susceptibility based on the results of planktonic cells. The morphology of the tested biofilms is known to be different. Generally, *S. aureus* biofilms are denser than those of *E. coli* [69].

Other authors studied the control of *S. aureus* and *E. coli* biofilms on stainless steel, concluding that the last ones are more susceptible to removal by five types of cleaning agents, food additives and other compounds [69]. In other studies, phytochemicals showed higher potential to prevent and control biofilms formed by Gram-negative bacteria compared to Gram-positive [18,32]. In a recent study of Abreu *et al.* [70], the effects of a glucosinolate at different concentrations was used against biofilms of *E. coli* and *S. aureus*. The percentages of biofilm removal 1 h after treatment with phenyl isothiocyanate were considerable for both biofilms. However, *E. coli* biofilms were inactivated at a higher extent than those from *S. aureus* [70]. This result proposes that the mechanisms of antimicrobial resistance known for planktonic cells cannot be used to infer on the antimicrobial resistance of biofilms. In fact, planktonic Gram-negative bacteria are known to be more resistance to antimicrobial agents acting on multiple targets of the cell, such as phytochemicals [63], than Gram-positive bacteria. This is attributed to the presence of an outer membrane in the Gram-negative bacteria that provides a protective barrier to the entry of antimicrobials.

### 2.4. Combination of the Selected Phytochemicals with Antibiotics

The use of dual combinations of antimicrobial drugs with positive *in vitro* interactions has become an important parameter to potentiate the therapeutic action of antibiotics. These combinations are expected to exert a synergistic effect or to reduce possible adverse side effects. The use of active compounds, such as phytochemicals, in conjunction with antibiotics could avoid the emergence of resistant variants that might otherwise arise during treatment [71]. Several phytochemicals have been analyzed for their action as resistance-modifying agents (RMAs), *i.e.*, compounds able to modify or inhibit bacterial resistance, increasing the efficacy of antibiotics to kill resistant bacteria [71]. Efflux pumps contribute to the resistance of bacteria by pumping out a wide variety of products: dyes, detergents and antibiotics [16]. The role of efflux pumps in bacteria has been related to the elimination of metabolites that are poisonous to the cell and in cell stress responses [72]. The association of antibiotics with phytochemicals can create a synergistic effect against resistant bacteria, creating new choices for the treatment of infectious diseases.

*S. aureus* genome reveals high potential multidrug efflux-pump-encoding genes [73]. Several efflux resistance mechanisms have been described for *S. aureus* such as QacA and NorA, which are multidrug transporters, and the more specific MsrA and TetK transport proteins [74]. 

In this study, the antimicrobial activity of several phytochemicals was tested in combination with three antibiotics. For these experiments, besides *S. aureus* CECT976, three different strains of *S. aureus* were tested: *S. aureus* RN4220, SA1199B, XU212. The strains are characterized by the presence of different efflux pumps: MsrA macrolide efflux protein, NorA MDR efflux protein and TetK efflux pump. Table 7 shows the diameter of the inhibition halos (mm) obtained with the antibiotics (ciprofloxacin, tetracycline and erythromycin) and the phytochemicals alone against the different *S. aureus* strains.

**Table 7:** Antimicrobial activity of antibiotics. The means (mm) ± standard deviation for at least three replicates are illustrated.

	Diameter of inhibition zone (mm)			
	*S. aureus* CECT 976	*S. aureus* XU212	*S. aureus* RN4220	*S. aureus* SA1199B
TET	41.5 ± 9.2	16.0 ± 4.2	42.5 ± 0.7	46.5 ± 2.1
ERY	37.5 ± 3.5	24.5 ± 4.9	22.0 ± 4.2	35.5 ± 3.5
CIP	40.5 ± 0.7	26.0 ± 2.8	31.5 ± 2.1	18.0 ± 1.4

According to Clinical Laboratory Standard Institute (CLSI) guidelines [75], *S. aureus* CECT 976 is considered susceptible to all antibiotics tested, *S. aureus* XU212, *S. aureus* RN4220 and *S. aureus* SA1199B are resistant to TET, ERY and CIP, respectively. The negative control performed with dimethyl sulfoxide (DMSO) in the preparation of phytochemical solutions presented no effects on bacterial growth (data not shown).

Tetracycline was the most effective against all the bacteria (except for *S. aureus* XU212), while erythromycin and ciprofloxacin had the lowest antimicrobial activity against *S. aureus* CECT 976/RN4220 and *S. aureus* SA1199B, respectively (*p* < 0.05).

Dual combinations of antibiotic-phytochemicals were performed. Antibiotic synergism occurs when the effects of combination of antimicrobials is greater than the sum of the effects of individual antimicrobials [76]. An additive effect of phytochemical combined with antibiotic may occur due to a double attack of both agents at different target sites of the cell [77]. Table 8 shows the classification of the combined application of tetracycline, erythromycin and ciprofloxacin with the phytochemicals according to Saavedra *et al.* [76].

**Table 8:** Classification of the effect of dual combinations of phytochemicals and antibiotics.

		7-HC	I3C	SA	SP
*S. aureus* CECT 976	TET	+	+++	+	−
	ERY	−	+++	+	++
	CIP	+	+++	+	−
*S. aureus* XU212	TET	++	+++	+++	+++
*S. aureus* RN4220	ERY	−	+++	+++	+++
*S. aureus* SA1199B	CIP	+	+++	+++	+++

(−)—Antagonist; (+)—Indifference; (++)—Additive; (+++)—Synergistic.

The combination of I3C with all the antibiotics showed synergistic effects against the four *S. aureus* strains tested. Synergistic activities were also verified when combining SA or SP with TET, ERY and CIP against *S. aureus* XU212, *S. aureus* RN4220 and *S. aureus* SA1199B. This reinforces the advantageous antimicrobial effect of phytochemical-antibiotic combinations. Only two combinations presented additive results: 7-HC-TET against *S. aureus* XU 212 and SP-ERY against *S. aureus* CECT 976. However, the results of combination of antibiotics with the phytochemicals also presented negative effects: the association of 7-HC-ERY was antagonist against *S. aureus* CECT 976 and *S. aureus* RN4220. Also, the combination of SP-TET or SP-CIP presented an antagonistic activity against *S. aureus* CECT 976. The other combinations presented indifferent effects.

Previous studies already demonstrated the synergistic potential of phytochemicals when combined with antibiotics against pathogenic bacteria [7,27,37,76,78]. The alkaloid piperine, in combination with ciprofloxacin markedly reduced the inhibitory concentration and the mutation concentration of ciprofloxacin for several *S. aureus* strains, including MRSA [78]. The combination of four antibiotics (ciprofloxacin, erythromycin, gentamicin and vancomycin) with some sesquiterpenoid increased their antimicrobial activity against *E. coli* and *S. aureus*, comparatively to the antibiotic/sesquiterpenoids single application [37]. The application of dual combination demonstrated synergy between the aminoglycoside streptomycin with gallic, ferulic and chlorogenic acids, allylisothiocyanate and 2-phenylethylisothiocyanate against *E. coli* and *P. aeruginosa* [76]. In other work [7], another isothiocyanate, phenyl isothiocyanate, showed a good efficacy against *S. aureus* strains when combined with CIP and ERY due to an additive effect. Synergistic interaction was observed by Biswas and Roymon [27], on combined administration of saponin with chloramphenicol to inhibit *E. coli* strains.

## 3. Experimental Section

### 3.1. Bacterial Strains

Two strains from the Spanish Type Culture Collection (CECT) were used in this study: *E. coli* CECT 434 and *S. aureus* CECT 976. *S. aureus* RN4220 containing plasmid pUL5054, which carries the gene encoding the MsrA macrolide efflux protein; *S. aureus* SA1199B, which over expresses the NorA MDR efflux protein and *S. aureus* XU212, which possesses the TetK efflux pump and is also a methicillin-resistant *Staphylococcus aureus* (MRSA) strain, were kindly provided by S. Gibbons (University College London, UCL) [74,79,80,81]. Prior to use, these strains at −80 °C were transferred onto Mueller-Hinton (MH, Merck Germany) agar plate, grown overnight, and inoculated into MH broth at 30 °C and under agitation (150 rpm). Also, *Chromobacterium violaceum* ATCC 12472 were distributed over the surface of Luria-Bertani (LB, Merck Germany) agar and incubated for 24 h at 30 ± 3 °C

### 3.2. Phytochemicals and Antibiotics

The phytochemicals used were: 7-HC, I3C, SA and SP. The concentration of phytochemical used for the several experiments corresponds to MIC and 5 × MIC. Regarding the phytochemicals, 7-HC, SA and SP belong to phenolic group and I3C belongs to glucosinolate group. These compounds were obtained from Sigma (Sintra, Portugal) and prepared in DMSO (Sigma, Portugal). CIP, ERY and TET were obtained from Sigma (Portugal) and prepared in DMSO. The antibiotics were applied in different concentrations according to CLSI [75]. Ciprofloxacin was tested at 5 µg/disc, erythromycin was evaluated at 15 µg/disc and finally, tetracycline was assessed at 30 µg/disc. The three antibiotics tested belong to three different antibiotics classes: quinolone, macrolides, tetracycline. After preparation, antibiotic stock solutions were stored at −4 °C.

### 3.3. Determination of Minimal Inhibitory Concentration and Minimal Bactericidal Concentration

The MIC of phytochemicals was determined by microdilution method in sterile 96-well microtiter plates according to CLSI [75]. Overnight cell cultures (14 h incubation) of *S. aureus* and *E. coli*, in the exponential phase of growth, were adjusted to a cell density of 1 × 10^6^ cells/mL and added to sterile 96-well polystyrene microtiter plates (Orange Scientific, Belgium) with the different phytochemicals in a final volume of 200 μL. The antimicrobial solutions did not exceed 10% (v/v) of the well. DMSO was used as negative control. No antimicrobial activity was detected by DMSO (data not shown). Plates were incubated for 24 h at 30 ± 3 °C. MIC corresponds to the concentration in which the final optical density (OD) was inferior or equal to the initial OD.

MBC of phytochemicals was determined by the drop method [9,18]. After measuring the MIC, the wells corresponding to the phytochemicals concentrations equal and above the MIC were added (10 μL) to plate count agar (PCA, Sigma, Portugal) plates. The maximum concentration tested for the phytochemicals was 50,000 µg/mL. The drops were drained along the plate. After 24 h at 30 ± 3 °C, the plates were analyzed and the MBC of each phytochemical, corresponding to the concentration which inhibited the growth of the bacteria, was recorded. All tests were performed in triplicate.

### 3.4. Determination of Zeta Potential

Zeta potential experiments were performed according to Simões *et al.* [40]. The overnight cultures of *E. coli* and *S. aureus* were centrifuged at 3777 g for 10 min and washed twice with sterile water. Cells suspensions (at a final concentration of 10^9^ cells/mL), prepared in sterile tap water, of *E. coli* and *S. aureus* were incubated with phytochemicals (at MIC) for 30 min at 30 ± 3 °C. Cells suspensions without phytochemicals were used as control. The zeta potential experiments were performed using a Malvern Zetasizer instrument (Nano Zetasizer, Malvern instruments, Worcestershire, UK). All experiments were carried out in triplicate at room temperature and were repeated at least at three different occasions.

### 3.5. Physicochemical Characterization of Bacterial Surface

The physicochemical properties were measured using the sessile drop contact angle method [82]. After overnight growth, the cells suspensions were washed with NaCl (8.5 g/L) and centrifuged (10 min at 3777 g) twice. Cells suspensions prepared with sterile tap water (OD_640nm_ = 0.2 ± 0.02) were incubated with phytochemicals (at MIC) during 30 min. The solutions were filtrated (0.45 µm, Whatman, United Kingdom) and placed in microscope slides. The contact angle was measured with 3 different liquids: water, formamide (polar) and α-bromonaphtalene (nonpolar) (Sigma, Portugal).

The measurement of contact angles was performed using a model OCA 15 Plus (Dataphysics, Filderstadt, Germany) video based optical contact angle measuring instrument, allowing image acquisition and data analysis. The degree of hydrophobicity of a surface is expressed as the free energy of interaction between entities of that surface (s), when immersed in water (w)—ΔG_sws_. ΔG_sws_ (mJ/m^2^) can be positive or negative according of the interaction between the surfaces. In the case of ΔG_sws_ > 0, the material is considered hydrophilic, because the interaction between the two surfaces is weaker than the interaction of each entity with water. In contrast, when ΔG_sws_ < 0, the interaction between the surfaces is stronger than the interaction of each entity with water and the material is hydrophobic. Hydrophobicity was evaluated after contact angles measurements, following the van Oss approach [83,84,85].

The degree of hydrophobicity can be calculated through the surface tension components of interacting entities, according to Equation (1).

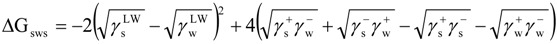
(1)
Where, γ^LW^ is the Lifshitz-van der Waals component of the surface free energy and γ^+^ and γ^−^ are the electron acceptor and donor, respectively, of the Lewis acid-base parameter (γ^AB^), being 
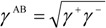
.

The analysis was performed at room temperature using the three liquids referred before. The surface tension components of liquids were obtained from literature [86]. Subsequently, three equations can be solved:

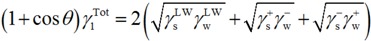
(2)
where θ is the contact angle and *γ*^TOT^ = *γ*^LW^ + *γ*^AB^.

These measurements were performed at least with 12 determinations for each liquid and microorganism.

#### Free Energy of Adhesion

The free energy of adhesion between the bacterial cells and polystyrene surfaces was calculated through the surface tension components of the entities involved in the process using the Dupré equation and the procedure described by Simões *et al.* [40]. The total interaction energy (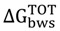) is studied by the interaction between one bacteria (b) and a substratum (s) that are immersed or dissolved in water (w) and is expressed by the interfacial tension components:

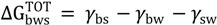
(3)

The thermodynamic theory of the interfacial tension of one system of interaction (for example, bacteria/surface—γ_bs_) can be defined by the following equations:

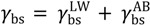
(4)

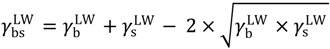
(5)

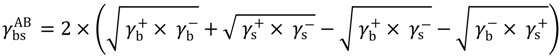
(6)

The two other interfacial tension components, γ_bw_ and γ_sw_, were calculated in the same way, which permits the assessment of thermodynamic energy of adhesion. The bacterial adhesion to the substratum can be favorable or is not expected to occur, according to the values of 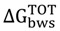 if are negative or positive, respectively [40].

### 3.6. Motility Assay

The motility assays were performed according to Borges *et al.* and Simões *et al.* [23,87]. Plates containing 1% tryptone, 0.25% NaCl and 0.25% or 0.7% (w/v) agar (Merck, Portugal) were prepared for swimming (for *E. coli*)/sliding (for *S. aureus*) or swarming motilities, respectively [88,89]. Phytochemicals at MIC concentration were incorporated in the growth medium after sterilize and cooling the medium, to avoid the deterioration. Overnight cultures of *E. coli* and *S. aureus* grown on LB broth (Merck, Germany) were adjusted to 1 × 10^8^ cell/mL and 15 µL of cell suspension were placed in the center of the plates. Then, plates were incubated at 30 °C and the diameter (mm) of the bacterial motility halos was measured at 24, 48 and 72 h [23]. All experiments were carried out in triplicate. The negative control was performed with DMSO.

### 3.7. Detection of Quorum-Sensing Inhibition

The biosensor strain *C. violaceum* CV12472 was grown overnight in LB broth at 30 ± 3 °C. MIC values were determined using the microdilution method, explained above. All the further experiments were performed at sub-MIC concentrations of phytochemicals [56].

Disc diffusion method was used to detect the inhibition of QS activity of the phytochemicals [90,91]. LB agar plates were spread with 100 µL of overnight culture of *C. violaceum* CV12472 (approximately 1.4 × 10^8^ CFU/mL). Sterile paper disks (6 mm diameter) (Oxoid, Spain) were placed in the plates and impregnated with various concentrations of each phytochemical (15 µL). DMSO and LB broth were used as controls. The plates were incubated at 30 ± 3 °C for 24 h, and then the inhibition of the pigment production around the disc (a ring of colorless but viable cells) was checked. Antimicrobial activity was indicated by the lack of microbial growth. Bacterial growth inhibition was measured as diameter 1 (d1) in mm while both bacterial growth and pigment inhibition were measured as total diameter 2 (d2) in mm. Thus, QSI, assessed by pigment inhibition, was determined by subtracting the diameter of bacterial growth inhibition (d1) from the total diameter (d2) (QSI = d2 − d1) [32,64].

### 3.8. Antibiotic-Phytochemical Dual Combinations Assay—Efflux Pumps Inhibition

This procedure was a modification from the Kirby-Bauer method and it has been applied in other studies [7,76]. Each phytochemical was added to MH agar (after autoclaved and cooled) yielding the final concentration desired and the medium was poured into 90 mm Petri dishes. Colonies of *S. aureus* strains were picked from overnight cultures (log phase cultures) and adjusted with NaCl (8.5 g/L) to match to 0.5 McFarland turbidity standards. The suspension was spread with a sterile cotton swap into Petri dishes (90 mm of diameter) containing 20 mL of MH agar. Sterile filter paper discs (6 mm in diameter) (Oxoid, Spain), impregnated with 15 µL of antibiotics, were placed on the agar plate seeded with the respective bacteria. Discs of ciprofloxacin, erythromycin and tetracycline (Sigma, Portugal) were used as positive controls and discs impregnated with DMSO were used as negative controls. The concentration of antibiotics used was according to CLSI [75]: ciprofloxacin—5 µg/disc; erythromycin—15 µg/disc; and tetracycline—30 µg/disc. The plates were incubated at 30 ± 3 °C for 24 h. After incubation, each inhibition zone diameter (IZD) was measured and analyzed according to CLSI guidelines [75]. All tests were performed in triplicate and the antibacterial activity was expressed as the mean of IZD (mm).

#### Classification of Dual Combinations

For the phytochemicals with antimicrobial activity, the effect of dual combinations of antibiotics and phytochemicals can be classified according [76]: Antagonism (−) – [inhibition halo—(antibiotic inhibition halo + phytochemical inhibition halo)/2] < 0; Indifference (+) – 0 ≤ [inhibition halo—(antibiotic inhibition halo + phytochemical inhibition halo)/2] < antibiotic inhibition halo or phytochemical inhibition halo; Additive (++)—antibiotic inhibition halo < [inhibition halo—(antibiotic inhibition halo + phytochemical inhibition halo)/2] < 2× antibiotic inhibition halo or phytochemical inhibition halo; Synergy (+++)—inhibition halo > 3× antibiotic inhibition halo or phytochemical inhibition halo. For this classification the highest inhibition halos caused by the antibiotic or phytochemical were used.

In the case of no antimicrobial effect of phytochemicals, the classification of the dual combination is different [71]: additive (++)—4 mm ≤ inhibition zone diameter combination—inhibition zone diameter most active agent) < 6 mm; synergistic (+++)—inhibition zone diameter combination—inhibition zone diameter most active agent ≥ 6 mm.

### 3.9. Biofilm Formation and Control in Sterile 96-well Polystyrene Microtiter Plates

Biofilms were developed according to the modified microtiter plate test proposed by Stepanović *et al.* [92]. For both bacteria, at least 8 wells of a 96-well polystyrene microtiter plate were filled with 200 μL of overnight batch cultures in MH broth (OD_620nm_ = 0.04 ± 0.02). The negative control wells were also placed on the plates, being sterile medium. The biofilms were formed in microtiter plates at 30 ± 3 °C for 24 h. After biofilm development, the content of wells was removed and the wells were washed three times with 200 μL of NaCl (8.5 g/L) to remove reversibly adherent bacteria. The phytochemicals were added to the wells at the MIC and 5 × MIC. The microtiter plates were incubated for 1 h. The remaining attached bacteria were analyzed using crystal violet (CV) and resazurin methods.

### 3.10. Biofilm Analysis

The mass quantification by CV method was based in previous studies [40,87,93]. Before (control wells) and after phytochemicals application, the inoculum in the wells was removed and the wells were washed with 200 μL of sterile water. Later, 250 μL of ethanol were loaded for 15 min to promote biofilm fixation. The supernatant was removed and the plates were air-dried. Subsequently, 200 μL of CV solution (Gram color staining set for microscopy, Merck, Germany) was added for 10 min to stain the fixed bacteria. After washing in water, the plates were dried and finally, the wells were loaded with 200 μL of acetic acid 33% (v/v) (Merck, Germany) to release and dissolve the stain. In order to analyze the biofilm, the OD of the solutions was measured at 570 nm using a microtiter plate reader (SpectraMax M2E, Molecular Devices, Norway). After obtaining the values of absorbance, the percentage of biomass removal was calculated.

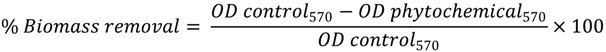
(7)
where OD control_570_ represents the optical density of the control at 570 nm, and OD phytochemicals_570_ is the optical density of the phytochemical at 570 nm.

The resazurin microtiter plate assay was performed to evaluate the metabolic activity according to Sarker *et al.* [94]. For the resazurin method, a commercially available resazurin solution (Sigma, Portugal) was used. The plates were loaded with 190 μL of sterile MH medium and 10 μL of resazurin solution (0.1 mg/mL). After 20 min of incubation in darkness and at room temperature, fluorescence (λ_ex_: 570 nm and λ_em_: 590 nm) was measured using the microtiter plate reader. After measuring the fluorescence, it is possible to calculate the percentage of metabolic inactivation.

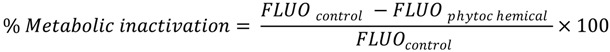
(8)
where FLUO_control_ represents the fluorescence intensity of biofilms not exposed to phytochemicals and FLUO_phytochemical_ represents the fluorescence intensity value for biofilms exposed to phytochemicals.

### 3.11. Statistical Analysis

The data was analyzed using One-Way Anova and the statistical program SPSS 21.0 (Statistical Package for the Social Sciences). The results were presented as the means ± standard deviation. Significance level for the differences was set at *p* < 0.05 and the calculations were based on confidence level equal or higher than 95%. 

## 4. Conclusions

In order to find new antimicrobial agents, plant products were studied as substituents or complementary products of antibiotics for which bacteria already acquired resistance. Therefore, in this work, the antimicrobial effect of selected phytochemicals—7-HC, I3C, SA and SP—was evaluated as well as their ability to control biofilms of two important pathogens, *E. coli* and *S. aureus*.

This study demonstrates that 7-HC and I3C are the most promising phytochemicals against *E. coli* and *S. aureus*. The 7-HC was one of the most effective phytochemicals tested against *E. coli* and *S. aureus* with a MIC of 800 and 200 µg/mL for *E. coli* and *S. aureus*, respectively. Regarding the biofilm control, the exposure of *S. aureus* biofilms to 7-HC at different concentrations produced significantly different percentages of inactivation (39 at MIC and 47 at 5×MIC). I3C was also effective against both bacteria with MIC of 800 and 400 µg/mL for *E. coli* and *S. aureus*, respectively. Dual combinations of all the antibiotics and I3C presented a synergistic effect against *S. aureus* resistant strains. Both phytochemicals (I3C and 7-HC) affected the motility and QS activity, which means that they can play an important role in biofilm prevention and interference with cell-cell interactions. The phytochemicals also demonstrated significant potential to reverse antibiotic resistance. However, in order to apply these phytochemicals with therapeutic/clinical purposes, further studies are required to ascertain their toxicity against mammalian cells and to confirm *in vivo* their efficacy and potential side effects.
